# Measurable residual disease in hairy cell leukemia: Technical considerations and clinical significance

**DOI:** 10.3389/fonc.2022.976374

**Published:** 2022-11-10

**Authors:** Tadeusz Robak, Paweł Robak

**Affiliations:** ^1^ Department of Hematology, Medical University of Łódź, Łódź, Poland; ^2^ Department of General Hematology, Copernicus Memorial Hospital, Łódź, Poland; ^3^ Department of Experimental Hematology, Medical University of Łódź, Łódź, Poland; ^4^ Department of Hematooncology, Copernicus Memorial Hospital, Łódź, Poland

**Keywords:** *BRAF*, cladribine, hairy cell leukemia, flow cytometry, immunohistochemistry, minimal residual disease, moxetumomab pasudotox, PCR

## Abstract

Hairy cell leukemia (HCL) is a rare type of chronic lymphoid leukemia originating from a mature B lymphocyte. A diagnosis of HCL is based on cytology, confirmed by multiparametric flow cytometry (MFC) studies using anti-B-cell monoclonal antibodies, together with a panel of antibodies more specific to HCL, such as CD11c, CD25, CD103 and CD123. Recently, the *BRAF* V600E mutation has been described as a disease-defining genetic event. Measurable residual disease (MRD) is defined as the lowest level of HCL cells that can be detected accurately and reproducibly using validated methods; as MRD negativity is associated with high rates of durable complete response, by clearing MRD, the long-term outcome may be improved in patients with advanced HCL. MRD is typically detected using bone marrow, and in some cases, peripheral blood; however, in HCL, discrepancies frequently exist between MRD results obtained from blood, bone marrow aspirate and core biopsy. Among the methods used for MRD detection, MFC appears to be a more sensitive technique than immunohistochemistry. Molecular tests are also used, such as real-time quantitative PCR for unique immunoglobulin heavy chain (IgH) gene rearrangements and PCR techniques with clone specificity for *BRAF* V600E. Clone-specific PCR (spPCR) is able to detect one HCL cell in 10^6^ normal cells, and is particularly suitable for patients found to be negative for MRD by MFC. Recently, the Hairy Cell Leukemia Consortium created a platform to work on a definition for MRD, and establish the optimal time point, tissue type and method for measuring MRD. This

## 1 Introduction

Hairy cell leukemia (HCL) is a rare type of chronic lymphoid leukemia originating from a mature B lymphocyte (1, 2). Its incidence is 0.3 cases per 100,000 individuals, and median age at diagnosis is 58 years. Approximately 1000 new cases of HCL are diagnosed each year in the United States (3). HCL is four times more common in men than women (4).

A diagnosis of classical HCL is based on morphological, characteristics of hairy cells and immunologic phenotype in multiparametric flow cytometry (MFC) and immunohistochemistry (IHC) in the trephine biopsy and the presence of BRAF^V600E^ somatic mutation (5). Anti-B-cell monoclonal antibodies (MoAb) such as CD19, CD20 or CD22, are used together with antibodies more specific to HCL including CD11c, CD25, CD103 and CD123. More recently, CD200 and LAIR1 were introduced as important markers of HCL (6). Classic HCL is characterized by mutation of the BRAF serine/threonine protein kinase (V600E) with an incidence of nearly 100% of HCL cases at diagnosis (7, 8).

Purine nucleoside analogues (PNA), pentostatin (deoxycoformycin, DCF) and cladribine (2-Chlorodeoxyadenosine, 2-CdA), are recommended for first-line treatment in classic HCL (9, 10). These agents induce durable and unmaintained complete response (CR) in more than 70% of patients, and the relapse rates are about 30% to 40% after 5 to 10 years of follow-up, with overall survival (OS) frequently longer than 20 years (11–13). Patients may expect a normal lifespan when treated with PNA, irrespective of their pretreatment history (13). While 2-CdA and DCF demonstrate similar efficacy and safety (14), 2-CdA is a more common choice than DCF due to its shorter treatment duration (15).

Although median time to relapse following 2-CdA treatment is 16 years, disease-free survival (DFS) and relapse-free survival (RFS) curves have not yet reached a plateau, suggesting that most patients who live long enough will eventually relapse. A recent multicenter analysis in Europe confirmed that 2-CdA used as frontline treatment in HCL patients permits disease control in a significant proportion of cases, given that more than 50% of treated patients require no further therapy. Good quality responses may be maintained for more than 20 years in up to 35% of patients (12).

Rituximab is an effective drug in HCL, especially when used in combination with other agents (16, 17). When rituximab was combined with 2-CdA in early relapsed HCL, CR was achieved in 89-100% of patients, with a 5-year progression-free survival (PFS) of 100% and a 3-year risk of relapse only 7% (16). Recently, several new drugs have been introduced for the treatment of patients with HCL (18). Among these, clinical trials have confirmed the anti-CD22 immunotoxin moxetumomab pasudotox (Moxe), BRAF kinase inhibitors (vemurafenib and dabrafenib), MEK inhibitors (trametinib and cobimetinib) and the Bruton’s kinase inhibitor ibrutinib as useful agents in the treatment of patients refractory to PNAs (19–24).

Measurable residual disease (MRD) is defined as the lowest level of neoplastic cells that can be identified using validated methods i.e. their detection below the level of conventional cytomorphology using more sensitive methods, including IHC, MFC, cytogenetics and molecular techniques (25–30). Several studies have indicated that the detection of MRD after therapy for HCL has prognostic value. In particular, clearing MRD may improve long-term outcome in patients with advanced disease (27). It has been shown that in patients treated with 2-CdA, the appearance of positive MRD in bone marrow (BM) may predict disease recurrence in most patients (25, 26). Clinical trials exploring the potential value of MRD evaluation in HCL patients treated with novel drugs, including monoclonal antibodies (MaAbs), immunotoxins and BRAF inhibitors, alone or in combination with other agents, are ongoing. This review presents the current state of knowledge on MRD in HCL, including methodology, clinical results and future directions.

## 2 Methods of MRD detection in HCL

Measurable residual disease (MRD) is becoming an important investigative tool in the clinical management of several hematologic malignancies, including forms of acute leukemia, chronic myeloid leukemia, chronic lymphocytic leukemia (CLL) and multiple myeloma. In many hematologic neoplasms, especially CLL, MRD has been indicated as a biomarker in clinical trials (31). In HCL, MRD is defined as the lowest level of leukemic cells that can be identified using validated methods (28). Currently, MRD detection in hematological malignancies is based on sensitive methods, such as identifying tumor-associated immunophenotypic characteristics by MFC, or evaluating specific genetic markers by PCR-based methods and next-generation sequencing. In HCL, MRD is evaluated in peripheral blood (PB) and BM aspirate or core biopsy. The presence of MRD should be determined in the context of the sensitivity of the used techniques and the ability of participating laboratories to accurately and reproducibly detect it. The sensitivity of each method is carefully specified (32). In particular, the lowest level of detectability (LLOD) and lowest level of quantitation (LLOQ) should be taken into account. For each illustrated analytical approach, the ranges of attainable LLOD and LLOQ should be carefully specified, since they vary remarkably both with technology and with time. It is widely believed that using 0.01%/10^-4^ as a threshold is less relevant in most hematologic neoplasms, and that future MRD analyses should use a lower LOD (preferable <0.001%/10^-5^) (32, 33). A consensus report on the potential application of MRD assessment in front-line and relapse settings and recommendations on the future role of MRD assessment in HCL has been recently developed by the International Group of Experts on Measurable Residual Disease in Hairy Cell Leukemia and should be published soon.

### 2.1 Immunohistochemistry

Several studies have performed immunohistochemical staining of BM core biopsy. Bengio et al. compared IHC with MFC with for MRD detection in HCL patients after therapy with 2-CdA (29). The procedure used the CD20 monoclonal antibodies L26 and DBA.44 to detect MRD by IHC, and CD20, CD22, CD25, Sig, CD11c and CD103 Mo Abs by MFC. The definition for positive MRD was 1–10% CD20/DBA44 scattered or clustered cells with tricoleukocyte morphology for IHC, and any expression of CD11c/CD25/CD103 in the BM or PB for FC. The MRD positivity rate for IHC was 46% compared with 64% for MFC, suggesting that MFC is a more sensitive technique than IHC.

A recent study by Gupta et al. evaluated the potential of two IHC staining assays to detect HCL involvement in core biopsies (30). Bone marrow IHC was performed using PAX5/CD103 and PAX5/tartrate-resistant alkaline phosphatase (TRAP) dual IHC stains. The sensitivity of the dual IHC stains was found to be 81.4%, positive predictive value was 100% and negative predictive value 81.7%. Simultaneously-performed MFC found the dual IHC allowed the detection of HCL cells even when the disease burden was as low as 0.02% of all identified lymphoid cells. In this study, some of the patients found to be positive with dual IHC staining were also negative for morphologic evidence of disease based on CD20 and H&E stains, suggesting that MRD detection by dual IHC stains is more sensitive than single IHC stains. However, one of the cases with an extremely low disease burden was found to be negative by dual IHC staining, and positive by MFC.

### 2.2 Flow cytometry

In several hematologic malignancies, the most commonly-used procedure for detecting MRD is flow MFC. In recent years, significant progress has been made in the methodology and interpretation of MFC results. Flow cytometry evolved from a basic (4-color) method to the modern MFC multidimensional cell analysis with ≥6–8 colors (31). In MRD detection, MFC allows the simultaneous recognition of several phenotypic markers (usually 6–8 antigens), and the capacity to analyze large numbers of cells in only a few minutes. The method now offers similar sensitivity to the most sensitive molecular techniques. In HCL, MRD detection by MFC is usually performed by immunophenotyping based on antibodies reacting with antigens characteristic for HCL: CD19, CD20, CD22, CD25, CD79a, CD11c, CD103 and surface immunoglobulin (32). The use of MFC with these markers has sensitivity, typically in the range of one HCL cell per 100 000 cells (1x10^-5^) (32). MFC is the most commonly-used method, as it is the most practical and informative. In 2012, the EuroFlow consortium, presented novel consensus protocols, for standardization of MFC in the diagnosis of hematologic diseases (34). Based on these guidelines, recently specific procedural recommendations for sample collection. An adequate BM or PB sample (1-2 ml) and an extensive antibody panel with backbone and lineage markers is needed is needed for MFC analysis of MRD. In addition, millions of clean CD45+ cell events should be acquired to ensure adequate LLOD and LLOQ levels (35). More recently, the International Group of Experts on Measurable Residual Disease in Hairy Cell Leukemia developed specific guidelines for evaluation of MRD in HCL (manuscript submitted). These specify that high-quality first-pull BM aspirate samples are required for MFC, as hemodilution can prevent the correct quantification of MRD in the BM, and to a greater degree than in other leukemias, mainly due to the limited PB involvement by HCL cells.

### 2.3 Molecular methods

Originally, molecular methods based on qualitative PCR and real-time quantitative PCR (RQ-PCR) were used for MRD detection (28, 36–38), while some studies have used RQ-PCR for unique immunoglobulin heavy chain (IgH) gene rearrangements. Recently, more advanced PCR techniques have been introduced, including droplet digital PCR and whole-genome sequencing methods known as next generation sequencing (NGS). Amplification using consensus V primers (most commonly for the framework 1-3 regions of IgH, [cpPCR]) has a sensitivity ranging from 1x10 ^-4^ to 1x 10^-5^ (32); however, PCR methods with clone specificity offer greater sensitivity (1 x 10^-^ **^6^)**, and some centers use PCR for *BRAF* V600E mutation (29).

In a study of previously-treated HCL patients, Sausville et al. found PB MFC (CD19, CD22, CD103, FMC7, CD23, CD19, CD20, CD11c, CD25, CD45, CD4, CD8, CD3, CD5, CD7, CD2) to be more sensitive than clonal analysis using consensus primer PCR (cpPCR) for the heavy chain gene (37). The results indicate that 31% of the MFC-positive cases were found to be negative by cpPCR, and only 1% of the cpPCR-positive cases were negative by FC. To improve the sensitivity of detection of MRD, consensus primers assay with CD11c sorting can be used (28). This method, called real-time quantitative PCR, was able to detect one HCL cell in 10^6^ normal cells.

Arons et al. compared the sensitivity of MFC and RQ-PCR assay based on patient-specific primers and probes for the IgH gene rearrangement, for detecting MRD (38). In this study MRD assessed by MFC was compared with consensus primer PCR (cpPCR) and splinkerette PCR (spPCR) after therapy with the recombinant immunotoxin BL22 (38). The MRD positivity rates were found to be 74% by FC, 55% by cpPCR, and 98% by spPCR. Moreover, quantitative levels of spPCR correlated with disease status. These findings suggest that spPCR may be most useful once negativity for MRD has been established by MFC. RQ-PCR was more sensitive than MFC and the quantified relative level of MRD correlated with disease status. This study suggests that patient-specific RQ-PCR is a very sensitive test for MRD in HCL patients and could be used to monitor maximal response in patients treated with antileukemic drugs.

Digital droplet PCR (ddPCR) has recently been applied for the detection of BRAF^V600E^ mutation in HCL (39). This is a molecular method that allows quantification of DNA mutations and the detection of the *B-RAF* V600E mutation and MRD status. Guerrini et al. used the ddPCR in retrospective study of 47 HCL patients including 27 with classic HCL, two with HCLv and 18 with splenic marginal zone lymphoma (SMZL) (12, 39). The study found the sensitivity of dd-PCR to be about half a logarithm superior to QT-PCR (5 × 10^-5^ vs. 2.5 × 10^-4^). Moreover, the specificity of the dd-PCR was similar to QT-PCR in classic HCL. The authors suggest that dd-PCR can be a useful method in the detection and monitoring of MRD in HCL patients. At the end of the treatment, 33% of patients in CR were found to be still MRD-positive after 12 months by dd-PCR, and 28% by QT-PCR. These findings suggest that dd-PCR may be more sensitive than quantitative PCR and can be useful for detecting MRD in HCL. In a similar study, Broccoli et al. measured *BRAF* V600E burden by ddPCR in PB and/or BM in 35 HCL patients at diagnosis, relapse, and CR (12). In PB, the mean fractional abundance values were 12.26% at diagnosis, 16.52% at relapse and 0.02% at CR, with the corresponding values in BM being 23.51%, 13.96%, and 0.26%. In addition, four of six patients evaluated at response were molecularly negative for BRAF^V600E^ in PB. The mean fractional abundance in PB evaluated in 14 patients with long lasting CR was 0.05%, and 10 were *BRAF* V600E negative, indicating that some patients in CR demonstrate a molecular CR. These results indicate that ddPCR for BRAF^V600E^ is a useful method for monitoring MRD in classic HCL.

## 3 Minimal residual disease in clinical trials

Clinical trials exploring the potential for purine analogs, MoAbs, immunotoxins and BRAF and MEK inhibitors to eradicate MRD have been performed in HCL patients. The results have been published over recent decades (**Tables 1**, **2**).

**Table 1:** Clinical studies with MRD evaluation in patients with HCL treated with purine nucleoside analogs.

Study regimen	Phase of the study/Disease status	Number of patients	Response (OR/CR)	Method of MRD evaluation	MRD negativity	PFS	References
2-CdA 0.1 mg/kg/d by continuous i.v. for 7 days	Phase 2/15 previously untreated	33, 31 evaluable.	100**%**/77**%**	IHC with MoAb B-ly 7 in L26 and MB2, and UCHL-1	19 from 24 in CR (80%)	NR	Tallman et al., 1992, Hakimian et al., 1993 (31, 40)
2-CdA 0.07 mg/kg/d for 7 days	Retrospective/relapsed	14	100**%**/78**%**	IHC with MoAb B-ly 7	0%	After median follow-up 19.3m 9 pts in CR and 2 relapsed (at 22 and 27 m)	Konwalinka et al., 1995 (41)
2-CdA 0.14 mg/kg s.c. x 5 days	Retrospective	17		IHC with CD45, CD20, DBA.44, and CD3	Gr 1: MRD <1% - 7 pts; Gr 2: MRD 1% to 5% - 6 pts, Gr 3: MRD>5% - 4 pts	Gr 1- all in CR at 55.4 mfollow-up;Gr 2 – 3 pts in CR after 77.9, 63.8, and 108.0 months;GR 3 -3pts relapsed	Mhawech-Fauceglia 2006 (42)
2-CdA 0.085 to 0.1 mg/kg per day x 7 days	Retrospective/untreated	19 with long CR selected from 358	100**%**/100**%**	FC for CD103, CD11c, and CD25) on BM aspirates in 17 or IGH-PCR	47%	Median time from 2-CdA, 16 years	Sigal et al., 2010 (43)
2-CdA (5.6 mg/m^2^ for 5 days) followed by R 375 mg/m^2^	Phase 2/untreated 11, relapsed 2	13	100**%**/100**%**	IGH-PCR assay and FC	FC negative in 22/28 (79%)pts and PCR in 19/27 (70%) after R,	Median response duration 9 m (4-16 m).	Ravandi et al., 2006 (44)
R 375 mg/m^2^/wk x 4 after pretreatment with 2-CdA	Phase 2/pretreated with 2-CdA	8 (2 CR, 4 PR, 2 no response)	100**%%**/100**%%**	IGH-PCR	100% 1 yr after the end of R treatment.	NA	Cervetti et al., 2004 (45)
2-CdA (5.6 mg/m^2^ for 5 days) followed by R 375 mg/m^2^	Phase 2/untreated	36 (5 with HCLv)	100**%**/100**%**	IGH-PCR assay and FC in PB and BM	76% and64%	Mefian not been reached (range,1+-63+ m	Ravandi et al., 2011 (36)
2-CdA (5.6 mg/m^2^ for 5 days) followed by R 375 mg/m^2^	Phase 2/untreated 59, relapsed 14, HCLv 7	80	CR untreated100%, relapsed 100% and 86%	Multiparameter FC at the time of response evaluation	94%	5-Year FFS: untreated 95%, relapsed 100% and HCLv 64%,	Chihara et al., 2016 (16)
2-CdA 0.15 mg/kg i.v./d x days 1-5 + R 375 mg/m^2^concurrent vs delayed	Phase 2/untreated	68	100**%**/100**%%** vs 100**%**/88**%**	FC in PB/BM and BM immunohistochemistry.	97 vs 24	Median ?94 vs 12	Chihara et al., 2020 (17)
Pentostatin 4 mg/m^2^every 2weeks until CR	Phase 1/relapsed	23	100**%**/100**%**	FC in PB and BM frozen sections using a panel of antibodies, CD11c, CD25, CD103 and HC2,	57% in BM,96% in PB	Median 59 m	Matutes et al., 1997 (46)
Pentostatin 4 mg/m^2^ every 2 weeks for 5 to 25 (median 13) courses	Phase 3/untreated	27	100**%**	Immunohistochemistry with CD20 and DBA.44 antbodes	7/27 (26%)	4/7 patients (57%) with MRD after DCF relapsed at 18, 21, 44, and 59 m after CR ad 0/20 without MRD	Tallman et al., 1999 (40)
Bendamustine 70 or 90 mg/m^2^ for 2 days + R 375 mg/m^2^ days 1 and 15 plus for for six cycles at 4-week intervals.	Phase 2/relapsed	12	100**%**/50**%** for 70 mg/m^2^ vs 67**%** for 90 mg/m^2^	FC in PB/BM and BM immunohistochemistry with L26, MB2, and UCHL-1 antibodies.	67% of CRs for 70 mg/m^2^ vs 100% of CRs for 90 mg/m^2^	31 months for patients in CR	Burotto et al., 2013 (47)

2-CdA, 2-chlorodeoxyadenosine, cladribine; BM, bone marrow; CR, complete response; DCF, deoxycoformycin, pentostatin;FC, flow cytometry; HCL, hairy cell leukemia; HCLv, HCL variant; IGH-PCR, immunoglobulin heavy chain gene rearrangements by consensus-primer polymerase chain reaction; ICH, immunohistochemistry; MoAb, monoclonal antibody; MRD, minimal residual disease; NA, not available; NR, not reported; PB, peripheral blood; OR, overall response; PCR, polymerase-chain-reaction; RFS, relapse-free survival; RQ-PCR, quantitative PCR.

**Table 2:** Clinical studies with MRD evaluation in patients with HCL treated with novel agents.

Study regimen	Phase of the study/Disease status	Number of patients	Response (OR/CR)	Method of MRD evaluation	MRD negativity	PFS	References
BL22 3 to 50 microg/Kg every other day x 3 doses.	Phase 1/relapsed	31	80**%**/60**%**	FC and consensus primers PCR	94% by FC and 100% by PCR	36 months for patients in CR	Kreitman et al., 2005 (48)
BL22	Phase 1 &2/relapsed	10	60**%/**/60**%**	FC and patient specific RQ-PCR	10% by RQ-PCR	NR	Arons et al., 2006 (38)
Moxe 32 - 50-µg/kg every other day for 3 doses in 4-week cycles	Phase 1 and extension/relapsed	33	88**%**/**%**64	FC in BM aspirate	33%	62.8m in MRD-negative and 12.0 in MRD-positive patients	Kreitman et al., 2012, 2018 (17, 48)
Moxe 40-µg/kg every other day for 3 doses in 4-week cycles	Phase 3/relapsed	80	75**%**/41**%**	FC in PB/BM and BM immunohistochemistry	34%	Median 71.7 months.	Kreitman et al., 2018, 2021 (19, 49)
Vemurafenib 960 mg bid x 16 -18 weeks	Phase 2/relapsed	54	100**%**/38**%**	Immunohistochemistry	0	1-Year PFS - 73%; median RFS 9 months	Tiacci et al., 2015 (19)
Vemurafenib 960 mg, twice daily for 8 weeks + R 375 mg/m^2^ for 8 doses over 18 weeks	Phase 2/relapsed	30	100**%**/87**%**	Allele-specific DNA PCR for *BRAF* V600E (sensitivity, ≥0.05% mutant copies)	65%	3-year PFS – 78%;	Tiacci et al., 2021 (50)
Vemurafenib 960 mg bid + Obinutuzumab 1000mg IV on days 1, 8, and 15 of m 2, and day 1 of m3 and 4.	Phase 1/untreated	9	100**%**/100**%**	Digital PCR for BRAFV600E	100%	9.7 m ongoing	Park et al., 2019 (51)
Dabrafenib 150 mg bid for 12 weeks.	Phase 2/relapsed	10	80**%**30**%**	Immunohistochemistry	0	7-60.5m	Tiacci et al.2021 (19)
Dabrafenib 150 mg bid + Trametinib 2 mg/daay until unacceptable toxicity or progression	Phase 2/relapsed, refractory	43	78**%**/49**%**	FC in PB and BM aspirates	15%	1-Year PFS - 98%	Kreitman et al., 2018 (51)
Ibrutinib 420 mg or 840 mg/day until unacceptable toxicity or progression	Phase 2/relapsed	37	73**%**/19**%**	FC in PB and BM aspirates and and BM immunohistochemistry	3 (8%)	3-Year PFS - 73%	Rogers et al., 2021 (24)

2-CdA, 2-Chlorodeoxyadenosine, cladribine; BM, bone marrow; CR, complete response; DCF, deoxycoformycin, pentostatin; FC, flow cytometry; IGH-PCR, immunoglobulin heavy chain gene rearrangements by consensus-primer polymerase chain reaction; ICH, immunohistochemistry; MoAb, monoclonal antibody; MRD, minimal residual disease; NR, not reported; PB, peripheral blood; OR, overall response; PCR, polymerase-chain-reaction; RFS, relapse-free survival; RQ-PCR, quantitative PCR; dabrafenib (150 mg twice daily) and trametinib (2 mg once daily) until unacceptable toxicity, disease progression, or death.

### 3.1 Purine nucleozide analogs

Clinical studies have evaluated MRD in patients with HCL treated with cladribine and pentostatin, used alone or in combination with rituximab, and the results are available.

#### 3.1.1 Cladribine

An early study performed by Konwalinka et al. evaluated MRD in 11 HCL patients in CR after treatment with 2-CdA (41). MRD was detected by IHC staining with the monoclonal antibody (MoAb) B-ly 7 and B-Ly7 (CD103). In all patients, MRD was found to range from 0.1 to 7.5% (median 0.65%). At a median follow-up of 29 months (median 19.3), nine patients remained in CR, while two relapsed 22 and 27 months from the end of 2-CdA therapy. MFC analysis of HCL cells was also performed in BM aspirates and PB using MoAbs, Leu-12 (CD19) and LeuM5 (CD11c) double-staining. In five of 10 cases, no hairy cells could be detected in the BM aspirates. In addition, no hairy cells were detectable in PB in six partly-different cases; however, hairy cells were identified in BM biopsy by B-ly 7 immunostaining (ranging form 0.1 to 7.5%). In other studies on patients treated with 2-CdA, by IHC was used to evaluate MRD in BM biopsies with the B-lineage antibodies L26 and MB2 (26, 52). In addition, BM core biopsies from 34 patients with HCL were studied before and three months after 2-CdA treatment, based on L26 (CD20) and MB2, and a T-lineage antibody, UCHL-1. Five of the 24 (21%) patients in hematologic CR were found to demonstrate MRD. Among 19 patients evaluated at one year, only one additional patient was found to be positive by immunostaining alone (26). In a longer observation, BM biopsies from 39 patients in CR after a single course of 2-CdA were evaluated by IHC with anti-CD45RO, anti-CD20 and DBA44 staining (25). Patients with detected MRD had a higher probability for disease progression than those without MRD (P=0.016) indicating that IHC evaluation of MRD has prognostic value (40).

Mhawech-Fauceglia et al. evaluated the correlation between the level of MRD and clinical outcome in patients treated with 0.14 mg/kg 2-CdA in subcutaneous bolus injections for five days (42). Conventional histologic examination and IHC were performed on sections of BM stained with CD45, CD20, DBA.44, and CD3 MoAbs in 17 patients with a median follow-up of 55.4 months. The patients were divided into three groups based on MRD level. Group 1 (seven patients) had MRD levels below 1%, and the patients remained in CR throughout the follow-up. In group 2 (six patients) MRD levels ranged from 1% to 5%; of these, three patients remained in CR at 77.9, 63.8, and 108.0 months. Group 3 (four patients) had MRD level above 5%; three patients in this group relapsed at 11.3, 12.1, and 29.6 months. This study further confirms that quantitative assessment of MRD has prognostic value and can predict dsease relapse. Ellison et al. determined MRD in HCL patients with CR using immunohistochemical staining for L26 and DBA.44 in BM biopsies (52). The study evaluated 154 BM biopsies from 42 patients between three months and 25 months after treatment with 2-CdA. Using this method, 91% of the biopsies were found to include DBA.44-positive cells, while 48% samples indicated HCL cells based on morphologic evaluation. Importantly, similar results were obtained over the 25-month follow up. This study indicated that immunomorphological analysis is a more sensitive technique for detecting HCL cells than morphology alone.

A study of 358 patients at the Scripps Clinic database by Sigal et al. identified 19 patients with residual MRD in long-lasting continuous hematologic CR after a single 7-day course of 2-CdA based on evaluable BM tissue specimens (43). Of this group, MRD was evaluated by multiparameter FC analysis based on CD103, CD11c and CD25 from the BM aspirates of 17 patients. Nine of the 19 (47%) patients had no evidence of MRD, seven (37%) had MRD and three (16%) had morphologic evidence of HCL.

#### 3.1.2 Cladribine plus rituximab

Cladribine combined with rituximab is more effective than 2-CdA alone in eliminating MRD in classic HCL and HCLv (16, 17, 44). Rawandi et al. treated 13 patients (two relapsed and 11 previously untreated) with 5.6 mg/m^2^ 2-CdA i.v. for five days, followed by eight weekly doses of rituximab (375 mg/m^2^) (44). All patients obtained a CR. MRD was assessed in PB and BM by immunoglobulin heavy chain (IgH) PCR assay using framework-1, -2, and -3 primer and FC assay with a four-color panel of antibodies. MFC confirmed MRD in 11 patients one month after 2-CdA therapy; however, negative MRD was observed in 12 of 13 patients after rituximab treatment. PCR assay confirmed MRD in five of 11 evaluable patients one month after 2-CdA therapy, and this became negative in 11 of 12 evaluable patients after rituximab. No patients have relapsed, with a median follow-up of 14 months (range, 6-16 months).

A subsequent study based on 31 patients with classic HCL and five with HCL variant (HCLv) evaluated a regimen comprising 5.6 mg/m^2^ 2-CdA for five days, followed one month later with 375 mg/m^2^ rituximab once a week for eight weeks (36). MRD was evaluated in BM after the end of rituximab treatment. Complete MFC and PCR. In most patients, MRD was also assessed by consensus primer PCR. MRD evaluated by MFC in BM was positive in 22 (85%) of 26 patients one month after treatment with 2-CdA, and this value became negative in 22 (79%) of 28 patients following treatment with rituximab. Consensus primer PCR testing identified positive MRD in 13 (54%) of 24 evaluable patients after treatment with 2-CdA, but negative MRD in 19 (70%) of 27 evaluable patients after completion of rituximab treatment. MFC evaluation failed to detect MRD in most patients over a longer follow–up. It was found that PB and BM demonstrated similar results for residual HCL in 23 (82%) patients, including 10 positive and 13 negative results. In the remaining five patients MRD was positive in the BM and negative in PB. These results may indicate that PB is less sensitive for MRD assessment than BM.

In a phase 2 study, Chihara et al. evaluated the efficacy of 2-CdA followed by rituximab in 59 patients with untreated HCL, 14 with relapsed HCL and seven with HCL variant (HCLv) (16, 17). Cladribine was given at a dose of 5.6 mg/m^2^ daily for five days, followed by 375 mg/m^2^ rituximab once weekly for eight weeks, one month after 2-CdA administration. MRD was evaluated by MFC at the time of response evaluation. The CR rate was 100% in patients with untreated and relapsed HCL and 86% in those with HCLv. Failure-free survival (FFS) at five years for each group was 95%, 100% and 64%, respectively. Negative MRD after treatment was achieved in 94% of the patients. Only 11 (14%) previously-untreated patients demonstrated MRD-negative disease after 2-CdA alone. However, no patients with relapsed disease or HCLv achieved negative MRD after 2-CdA monotherapy. Importantly, in most patients, positive MRD during the follow up did not result in clinical relapse.

Cervetti et al. analyzed the eradication of MRD with four cycles of rituximab in 10 HCL patients after pretreatment with 2-CdA (45). After treatment with 2-CdA, two patients were in CR, six in partial response (PR) and two without response. Median time from the end of 2-CdA treatment to rituximab infusion was 5.7 months. Rituximab was given at a dose of 375 mg/m^2^/week for four doses. Two months after the end of anti-CD20 therapy, all evaluated patients were in hematological CR. PCR with two consensus primers was used for MRD evaluation. Rituximab increased the percentage of molecular remission to 100% one year after the end of treatment. All patients but one showed MRD levels lower than those found before rituximab treatment. Recently, Chihara et al. presented the results of a long-term randomized study evaluating the effectiveness of combined rituximab and 2-CdA therapy in the elimination of MRD (17). Previously untreated patients with classic HCL were randomized to 2-CdA at a dose 0.15 mg/kg for five days, with eight concurrent weekly doses of 375 mg/m^2^ rituximab from day 1 (CDAR), or delayed rituximab started at least six months after detection of MRD. MRD was evaluated in PB or BM using FC, and BM immunohistochemistry. Six months after treatment, CR rates were 100% for CDAR versus 88% for 2-CdA monotherapy (*P* =0.11). In addition, MRD negativity rates were 97% versus 24% in BM (*P* <.0001) and 100% versus 50% in PB (*P* < 0.0001). At eight years median follow-up, undetectable MRD in CDAR group was 94% versus 12% in the delayed rituximab arm. However, 12 patients in the delayed rituximab arm were MRD negative at the end of rituximab administration were restaged between 6 and 104 (median, 78) months later. These results confirm that combined 2-CdA and rituximab therapy demonstrates high activity in achieving long-lasting MRD elimination in previously-untreated HCL patients, and is more effective than delayed rituximab use after 2-cdA monotherapy.

### 3.2 Deoxycoformycin

Matutes et al. investigated MRD in 23 classic HCL patients in CR after treatment with deoxycoformycin (DCF, pentostatin) (46). MRD was detected in PB and BM by immunophenotyping based on a panel of four antibodies specific for HCL cells: CD11c, CD25, CD103 and HC2. MRD was detected in 10 of 23 patients (43%) including seven in BM, one in PB and two in both BM and PB. However, the MRD-positive and MRD-negative patients demonstrated similar disease-free survival (DFS) (*P*=0.8). Unlike some other studies, relapse could not be predicted by MRD results; this could be due to the sensitivity of the method used in the study.

Tallman et al. evaluated MRD in 39 HCL patients treated with 2-CdA and 27 patients treated with DCF (40). The patients treated with 2-CdA received one course of treatment at a dose of 0.1 mg/kg/day for seven days by continuous i.v. infusion. The patients treated with 4 mg/m^2^ DCF every two weeks received from 5 to 25 (median 13) courses. All patients were in hematologic CR (**Table 1**). The criteria for MRD used in this study comprised a lack of HCL cells by routine morphology of PB and BM core sections, the presence of CD20- or DBA.44-positive cells equal to or higher than the number of CD45RO-positive cells, and the detection of 50% of CD20- or DBA.44-positive cells morphologically consistent with HCL cells. Seven of 27 patients (26%) treated with DCF demonstrated MRD compared with five of the 39 (13%) treated with 2-CdA. Among the patients without detected MRD, no relapses were noted in the DCF group, and only three relapses were noted out of 34 (9%) in the 2-CdA group. In total, six of the 12 patients (50%) with detected MRD and three of 54 patients (6%) without detected MRD relapsed. In contrast to the Matutes study above (46), positive MRD was associated with a higher risk of relapse: the estimated 4-year relapse-free survival (RFS) was 55% for patients with MRD and 88% for patients without MRD (P= 0.0023).

### 3.3 Bendamustin

Bendamustine is an alkylating agent active in the treatment of lymphoid malignancies. It is also effective for the treatment of classic HCL and HCLv, when used in combination with rituximab (BR) (47, 53). This treatment was evaluated in 12 relapsed or refractory HCL patients (**Table 1**) (47). The patients received rituximab 375 mg/m^2^ on days 1 and 15 and bendamustine 70 mg/m^2^ or 90 mg/m^2^ on days 1 and 2, for six cycles every four weeks for six cycles. Overall response rate was 100% for 70 mg/m^2^ and 90 mg/m^2^ bendamustine, with three (50%) and four (67%) CRs in the respective groups. MRD was not detected in 67% and 100% of CRs, respectively, and all six patients without MRD were in CR from 30 to 35 months of observations. MRD was confirmed in BM biopsy by IHC with L26, MB2, and UCHL-1 antibodies.

### 3.4 Immunotoxins

Anti-CD-22 immunotoxins, especially BL22 and moxetumomab pasudotox (Moxe), have been extensively investigated in relapsed/refractory HCL (48, 49, 54–57). BL22 is a recombinant immunotoxin containing a truncated form of the bacterial toxin Pseudomonas exotoxin A (PE38) attached to an Fv fragment of an anti-CD22 monoclonal antibody RFB4 (55). In a phase 1 study, BL22 was evaluated in 31 patients with PNA-resistant HCL (**Table 2**); of these, CRs were obtained in 19 (61%) (48, 58). Of the 19 patients achieving CR with BL22, only two were confirmed to demonstrate MRD by MFC, and none were found positive by PCR. In a phase 2 study performed in 36 patients, the OR rate was 72% and CR rate 47% (**Table 2**) (48, 56, 57). Most patients achieving CR to BL22 did not indicate MRD by either PCR or FC. MRD was then evaluated in 10 patients from the phase 1 and phase 2 studies, taken before or after BL22 treatment using MFC and patient-specific RQ-PCR (38). RQ-PCR was positive in all 62 (100%) MFC-positive samples from 10 patients and in 20 of 22 (91%) MFC-negative samples from six patients. Moreover, the level of MRD quantified by RQ-PCR correlated with disease status and response to treatment.

Subsequently, Kreitman et al. reported the discovery of the second-generation immunotoxin Moxetumomab pasudotox (Moxe (49, 58, 59). Moxetumomab pasudotox is a recombinant immunotoxin that binds to CD22-expressing cells, followed by internalization of the drug-CD22 complex (55). The drug was active and well tolerated in phase 1 and 3 studies performed in relapsed/refractory patients with HCL. In addition, Moxe can eliminate MRD in a significant number of patients, translated into greater CR duration (49, 57, 59). In the phase-1/2 study, Kreitman et al. analyzed the significance of MRD eradication with Moxe in 33 HCL patients, including 12 from the phase 1 study and 22 from the extension cohort, receiving 50-µg/kg Moxe every other day for three doses in four-week cycles (**Table 2**) (49). MRD was detected by 8-color multiparametric approach on a 3-laser FACSCanto II based on cells coexpressing CD19, CD20, CD22, bright CD11c and monoclonal light chains. Among the 33 analyzed patients, the OR rate was 88% including 64% CR. CR duration was longer in the MRD-negative patients: median CR was 13.5 months in nine MRD-positive CRs and 42.1 months in 11 MRD-negative CRs (*P* < 0.001). In a phase 3 trial 80 patients were treated with Moxe, given at a dose of 40 µg/kg by intravenous (i.v.) infusion on days 1, 3, and 5 of a 28-day cycle (20). Treatment was continued for up to six cycles, or until CR with MRD negativity, disease progression or unacceptable toxicity. MRD was assessed by quantitative MFC analysis of PB or BM aspiration, and by IHC on BM biopsy. At a median follow-up of 24.6 months, overall CR was 41%, with 36% demonstrating durable CR with hematologic response longer than 180 days, and 33% CR longer than 360 days. Twenty-seven (82%) patients with CR (34% of all patients) were MRD-negative. Longer median duration of hematologic remission was noted in MRD-negative patients than in MRD-positive patients (62.8 m vs 12.0 m, respectively).

### 3.5 BRAF inhibitors

The BRAF kinase inhibitors vemurafenib and dabrafenib are effective drugs in patients with refractory and recurrent HCL, either when used in monotherapy, or in combination with CD20 antibodies or MEK inhibitors (**Table 2**) (8). In a phase-2 single-arm multicenter study performed in Italy and US, vemurafenib was given as a single drug, 960 mg twice daily for a median of 16 - 18 weeks (21). Overall response rates were 96% (25/26) after a median of 8 weeks in the Italian study and 100% after a median of 12 weeks (24/24) in the US study. Complete response rates were 34.6% (9/26) and 41.7% (10/24), respectively. However, MRD was detected in all patients with CR at the end of treatment, evaluated by IHC. Moreover, the median relapse-free survival (RFS) was only nine months after treatment discontinuation. Deeper remissions were obtained when vemurafenib was combined with rituximab (60, 61).

In a phase 2 trial performed in 30 patients with refractory or relapsed HCL, vemurafenib was administered at a dose of 960 mg, twice daily for eight weeks, in combination with rituximab (375 mg/m^2^) for eight doses in 18 weeks (60). MRD was detected in PB and BM aspirates by means of allele-specific DNA PCR for *BRAF* V600E with a sensitivity ≥0.05% mutant copies. The primary end point was CR at the end of planned treatment, which was achieved in 26 patients (87%). Moreover, undetectable MRD was achieved in 17 (65%) of the 26 patients in CR. MRD negativity correlated with longer survival without relapse.

In another phase 2 study, vemurafenib was combined with obinutuzumab in previously-untreated HCL patients (50). Vemurafenib was given at a dose of 960 mg twice per day for four months and obinutuzumab at 1000 mg.iv. on days 1, 8 and 15 of month 2, and day 1 of month 3 and 4. MRD negativity was detected by BRAFV600E using highly-sensitive digital PCR. A total of 11 patients have been enrolled, of whom nine have completed treatment. Seven patients achieved MRD negative CR and two patients PR at the end of treatment. However, both patients with PR at month 4 converted to MRD negative CR by month 7 and 10. All patients remained in remission with a median follow-up of 9.7 months.

Another BRAF inhibitor, dabrafenib, was evaluated in a pilot phase 2 study in relapsed/refractory patients (22). Ten patients, including two previously treated with vemurafenib, received dabrafenib at a dose of 150 mg twice daily for eight weeks. If no CR was obtained after eight weeks, patients received an additional four-week course. Eight patients (80%) responded, including three with CR (30%) and five with PR (50%). However, all patients with CR had detectable MRD by immunohistochemistry in the BM biopsy. The duration of response in patients with CR was 15.5, 14 and 60.5 months. Moreover, of the patients in PR, one in five had 42-month survival on dabrafenib. The combination of BRAF inhibitor dabrafenib and MEK inhibitor trametinib hence appears even more effective than dabrafenib alone in V600E-mutated HCL (22, 51, 62).

In a phase 2, open-label trial, 43 eligible patients with refractory HCL received a combination of dabrafenib and trametinib (51). Minimal residual disease status was detected by flow cytometry in both PB and BM aspirates. At the time of data evaluation, 35 patients (81%) remained on treatment. Among 41 patients, 32 (78%) responded, including 20 (49%) with CR. Six (15%) patients in CR had no detectable MRD while 14 (34%) CR were MRD positive. Twelve (29%) patients obtained a PR. At the data cut-off; 16 (50%) responses had lasted 18 months or longer and no patients had experienced a relapse.

### 3.6 Ibrutinib

B-cell receptor (BCR) signaling is involved in HCL pathogenesis (62). In preclinical studies, Bruton’s tyrosine kinase (BTK) inhibitor ibrutinib inhibited survival, proliferation and B cell receptor signaling in HCL cells (63). Recently, ibrutinib was evaluated in a phase 2 study in 28 patients with classic HCL and nine patients with HCL-v (24). Ibrutinib was administered at a dose of 420 mg daily in 24 patients and 840 mg daily in 13 patients, until HCL progression or unacceptable toxicity (22). MRD was assessed in each patient based on FC in the PB and BM. and IHC examination using specific markers for HCL in the BM. Response was 24% at 32 weeks, and 36% at 48 weeks. The OR rate was 54% at any time since starting ibrutinib, including seven patients with CR, 13 patients with PR and 10 patients with stable disease (SD). MRD was not detected in three patients. The response rates were similar in patients with classic HCL and HCL-v. The estimated 36-month progression-free survival (PFS) was 73% and the estimated 36-month overall survival (OS) was 85%. However, MRD was not evaluated in this study.

## 4 MRD in HCL variant

In 2008, the World Health Organization (WHO) distinguished a new variant of hairy cell leukemia (HCLv). It was subsequently included as a provisional entity within the spectrum of splenic B-cell lymphomas/leukemia, unclassifiable (64). In the 5th edition of the WHO Classification of Haematolymphoid Tumours, the new entity splenic B-cell lymphoma/leukaemia with prominent nucleoli (SBLPN) replaces the previous term HCL variant (65). The World Health Organization reported 810 new cases of HCLv each year in the United States (3).

HCL-v is characterized by leukocytosis with lymphocytosis, cytopenias without monocytopenia, and lymphoid cells of relatively large size with prominent nucleoli. A critical aspect of HCL-v diagnosis is an atypical HCL immunophenotype without CD25 expression, and lack of *BRAF* V600E mutation (66, 67). Leukemic cells strongly express pan-B-cell markers, including CD19, CD20, CD22 and FMC7. Surface immunoglobulin expression is strong, with CD5 and CD23 usually negative. In contrast to classic HCL, CD25 and CD123 are negative but CD11c is always positive and CD103 is positive in 2/3 of HCL-v cases. Moreover, in HCL-v, Annexin A1 expression is negative. Some patients have activating mutations in *MAP2K1*, a gene that encodes MEK1, a downstream component of the BRAF-MEK-ERK signaling cascade. While there is no genetic mutation diagnostic of HCL-v, genetic profiling efforts have identified potential therapeutic targets, such as *MAP2K1, KDM6A, CREBBP, ARID1A, CCND3, U2AF1* and *KMT2C*.

PNA treatment yields unsatisfactory results for HCL-v treatment (66, 67). However, greater effectiveness has been reported for the combination of rituximab and 2-CdA. Kreitman et al. treated 10 patients with 0.15 mg/kg 2-CDA on days 1–5, with eight weekly standard doses of rituximab (68). Nine patients (90%) achieved CR, compared with three out of 39 (8%) treated with 2-CDA alone. In eight patients, MRD negativity was achieved. The median duration of response to 2-CDA + rituximab was longer than that seen for first-line 2-CDA alone (72 months vs not reached, *P* = 0·004). Positive MRD was noted during the follow up, but this did not result in any clinically-relevant relapse. Visentin et al. report effective treatment of three previously-untreated elderly patients with combined bendamustine and rituximab (53). All patients achieved a CR with no evidence of MRD, indicated by the absence of leukemic cells according to post-therapy immunohistochemical (CD20 and CD22) staging and flow-cytometry marrow examination. All three patients were in CR after a median follow-up of 19 months.

Another treatment regimen active in HCL-v is moxetumomab pasudotox (19, 49, 59). In a phase 1 and phase 3 study including six patients with HCL-v, MRD was independently evaluated using immunohistochemical staining for the HCL/B cell antigens CD20, CD79a, Annexin A1,DBA.44, and PAX-5 and by flow cytometric analysis of peripheral blood and/or bone marrow aspirate, according to each site’ s procedures. Although most of the patients responded to the treatment, no separate details exist for the subgroup with HCL-v. Future treatment for HCL-v may include targeted therapies such as ibrutinib, trametinib, binimetinib and venetoclax, and potentially anti-CD22 chimeric antigen receptor T cell therapy (CART**)** (69–72). Recently ibrutinib was evaluated in 37 patients, including 28 with classic HCL and nine with HCL-v (24). The HCL and HCL-v patients demonstrated similar response rates and estimated 36-month PFS and OS scores; however, the study was not designed to evaluate difference between both diseases. Ibrutinib is currently not approved by the FDA in HCL and HCL-V. Patients with HCL-v do not have a BRAF mutation and cannot be treated with BRAF inhibitors.

## 5 Practical considerations and perspectives

In most studies performed in HCL, investigators were able to predict relapse in patients with hematologic CR and a positive MRD test. In addition, eradicating MRD leads to a better outcome, longer PFS or OS or even recovery (17, 25). However, some studies indicate MRD was positive in most patients treated with 2-CdA with very long follow-up (median 16 years) (43). The practical value of MRD monitoring currently remains unclear, as does the value of a positive test for MRD as a predictor of clinical relapse. In some studies, patients with positive MRD after treatment with 2-CdA can survive even 16-18 years without clinical relapse (43). In addition, some patients treated with 2-CdA remain MRD negative for a considerable time, and can be considered as cured. In the future, MRD evaluation can be useful in deciding whether to continue treatment to achieve deeper response, to prolong CR duration or even cure. The introduction of novel drugs, such as immunotoxins and BRAF inhibitors, or novel combination regimens, such as immunochemotherapy, can be used to eliminate persistent MRD in some patients and decrease the risk of relapse (26). MRD monitoring may also be a useful indicator of the efficacy of novel drugs, as it allows shorter follow-up than standard criteria like PFS or CR duration, as with CLL. Currently, MRD is easily detectable by MFC and molecular techniques, provided the right technical methods are applied. Moreover, in hematologic malignancies, MRD detection is currently managed by internationally-applied external quality assessment/proficiency testing schemes, which confirms that it has clinical utility besides controlled trials. Recent studies with novel drugs have demonstrated that CR can be achieved with undetectable MRD in increase time of response. Several assays can be used to detect MRD in HCL patients; however, while IHC analysis of BM specimens used to be more popular, more recent guidelines recommend the use of MFC and PCR methods for detecting the mutant *BRAF* V600E gene or consensus primers for *IGH* (26, 44, 60).

Currently, MFC and allele-specific PCR analysis for mutant *BRAF* are recommended for detecting MRD in HCL (73–75). For patients treated with anti-CD20 monoclonal antibodies, MRD should be evaluated with other antibodies, such as the use of other B-cell marker (CD79a) or HCL-specific markers (eg,VE1) for IHC staining (1, 44). Recently, the ISCCA protocol for standardized prospective monitoring of patients treated with anti-CD20 therapies has been developed (73). MFC and quantitative or digital PCR are significantly more sensitive than IHC, and these tests should be recommended in the future studies and clinical practice. While MFC can achieve a sensitivity below 1/100,000 (1x10^-5^), investigated cells (75), molecular methods can achieve 10^-6^. MFC can be also used for the detection of MRD in a BM aspirate or PB. However, consensus needs to be reached regarding minimal level of detection in MRD in HCL, i.e. from 0.1% to 0.001%. The optimal sample type used for MRD detection is BM, however PB is also sometimes used.

Bone marrow core biopsy offers an alternative sample but immunohistochemical methods have limitations and are difficult to quantify. MFC appears to be a more sensitive technique for detecting MRD than IHC. Molecular tests, such as real-time quantitative PCR for unique immunoglobulin heavy chain (IgH) gene rearrangements with consensus V primers, demonstrates sensitivity ranging from 1x10 ^-4^ to 1x 10^-5^, with even greater values being noted for PCR techniques with clone specificity for *BRAF* V600E (1 x 10^-6^). Clone-specific PCR (spPCR) is able to detect one HCL cell among 10^6^ normal cells, and appears most appropriate for use in patients negative for MRD by MFC. At present, standardization for MRD detection is unachievable due to lack of standards and different platforms, reagents and processing methods.

No study has determined the optimal timing of the MRD evaluation in HCL patients. It seems rational to assess MRD when evaluating the response to treatment. The Consensus guidelines recommend that after 2-CdA therapy, a BM biopsy should be performed for four to six months after drug administration, or later if response is delayed and continuing improvement observed (1). In patients treated with DCF, the BM biopsy should be performed after optimal clinical response, including normalization of PB parameters. A similar approach seems to be rational for novel agents, active in HCL. There is a need to standardize MRD assessment in HCL, as has been the case in other hematologic malignancies, including chronic myeloid leukemia, acute lymphoblastic leukemia and chronic lymphocytic leukemia.

An expert panel should reach a consensus regarding the minimal level of HCL cell detection, optimal time point for MRD measurement, optimal type of samples used for MRD detection and detection (BM, PB) and optimal methods used for MRD evaluation. Currently it is not possible to standardize the methods used in MRD detection in HCL due to lack of standards and the wide range of platforms, reagents and processing methods currently used in different centers. Although harmonization is possible using different platforms, reagents and processing methods, it is difficult in the case of rare diseases. The Hairy Cell Leukemia Consortium is the most suitable platform for working on a definition of MRD, and establishing the optimal time point, tissue type and methods to measure MRD in HCL.

## 6 Conclusions

Measurable residual disease is defined as the lowest level of HCL cells that can be detected accurately and reproducibly using validated methods. MRD negativity is associated with high rates of durable complete response and long-term outcome may be improved by clearing MRD in patients with advanced HCL. However, long-term observation is needed to confirm the clinical benefit of MRD-negative CR after front-line treatment. Methods used for MRD detection include MFC, IHC and molecular tests. In HCL, discrepancies commonly exist between MRD results in blood aspirate and core biopsy. Bone marrow core biopsy offers an alternative sample, but immunohistochemical methods have limitations and are difficult to quantify. In addition, any MRD detection program should incorporate quality assurance that can confirm the ability of participating laboratories to accurately and reproducibly detect MRD. Available data on the role of MRD in the management of patients with HCL are not unambiguous and at present, MRD monitoring in HCL cannot be recommended in clinical practice. In the coming years, MRD assessment should be standardized asis the case in other hematologic malignancies, including acute lymphoblastic leukemia and chronic lymphocytic leukaemia (CLL). The Hairy Cell Leukemia Consortium has recently created a platform to work on a definition for MRD, and to establish the optimal time point, tissue type and methods for measuring MRD in HCL. Their opinion on the value of MRD monitoring in HCL patients is expected soon.

## Author contributions

All authors listed have made a substantial, direct, and intellectual contribution to the work and approved it for publication.

## Funding

This work was supported by the grants from the Medical University of Lodz, Poland (No. 503/1-093-01/503-11-004 and 503/1093-1/503-11-003).

## Acknowledgments

We thank Edward Lowczowski from the Medical University of Lodz for editorial assistance.

## Conflict of interest

The authors declare that the research was conducted in the absence of any commercial or financial relationships that could be construed as a potential conflict of interest.

## Publisher’s note

All claims expressed in this article are solely those of the authors and do not necessarily represent those of their affiliated organizations, or those of the publisher, the editors and the reviewers. Any product that may be evaluated in this article, or claim that may be made by its manufacturer, is not guaranteed or endorsed by the publisher.
